# Effects of interspecific interaction-linked habitat factors on moose resource selection and environmental stress

**DOI:** 10.1038/srep41514

**Published:** 2017-01-27

**Authors:** Heng Bao, John M. Fryxell, Hui Liu, Hongliang Dou, Yingjie Ma, Guangshun Jiang

**Affiliations:** 1 Feline Research Center of Chinese State Forestry Administration, College of Wildlife Resources, Northeast Forestry University, 26 Hexing Road, Harbin, Heilongjiang 150040, P.R. China; 2 Department of Integrative Biology, University of Guelph, Guelph, Ontario, N1G 2W1, Canada

## Abstract

Resource selection of herbivores is a complex ecological process that operates in relation to biological or non-biological factors, which may affect the feeding and movement, and subsequently their spatial distribution and environmental stress. Here, we estimated moose (*Alces alces cameloides*) resource selection for habitat variables and the effect of interspecific interactions related to roe deer (*Capreolus pygargus bedfordi*) on its population distribution and environmental stress in the Khingan Mountain region of northeast China at local and regional scales. Different response patterns of moose resource selection, spatial distribution, and environmental stress to interspecific interaction-linked habitat factors were shown at the two scales. A general ecological chain, response of moose to interspecific interaction-linked habitat factors, was exhibited at the regional scale, and at the local scale, heterogeneous responses, linkages of habitat selection and environmental stress of moose population might be driven by different interspecific interaction patterns. Our study firstly suggested that moose resource selection, food availability, diet quality, population density and environmental stress indicators were impacted by interactions with the distribution of other sympatric herbivore species and showed differences in ecological response chains at various spatial scales. These findings are useful for sympatric herbivore assembly conservation, habitat quality monitoring and management.

Resource selection of wild animals is a fundamental driver for population distribution and dynamics in space and time^1^,^2^, which should fulfill their requirements for growth, survival and reproduction^3^. This is often related to food quality and abundance, habitat, shelter, potential predators^4^ and inter- or intra-population interactions^5^. Henceforth, resource selection of herbivores is also a complex ecological process in relation to biological or non-biological factors. Analyzing how, when and why they select particular resources are the central issues^6^. The distribution of population density is the fundamental driver of many ecological processes, including wild animal resource selection^7^, and the differences in local population densities can be expected to match variations in habitat factors^8^. Habitat quality also affects population distribution and functional responses of large herbivores during resource selection^9^. A habitat may not contain an adequate combination of resources and the time spent within different habitat types might be influenced by the availability of resources^10^. Hence, wild animal diet composition may exist with geographical variation^11^, which may also lead to differences in animal resource selection.

Moose is typically a large browser and representative of the fauna in the frigid-temperate zone. In China, moose distribute only in the Greater Khingan mountain range and part of the Lesser Khingan mountain range in northeast China, where the herbivores’ large predator (specifically the Amur tiger, *Panthera tigris altaica*) had disappeared. In the absence of large predators or when the vulnerability is low, the location of moose feeding signs should be at the plants with high nutrient qualities^12^. Protein is widely known for a limiting nutrient indicator for wild animals and fecal nitrogen is a noninvasive indicator of diet quality and physical condition for large herbivores^13^,^14^. Facing the stress of psychology or physiology, glucocorticoids quickly mobilize glucose *in vivo*^15^,^16^, so fecal cortisol might also be an indicator of wild animal environmental stress. Interactions among populations or individuals also influence animal resource selection, and interspecific competition among sympatric herbivores is expected to be the most common interspecific interaction pattern in the absence of predators^5^. Furthermore, interspecific interaction has the potential to influence sympatric species distribution, habitat use and co-occurrence patterns^17^,^18^. In addition, the strength of interspecific interaction is likely affected by landscape features or human disturbances^18^,^19^. Competitive interactions for space and habitat between in coexistence similar species were relatively low when viewed at coarse spatial scales, while the opposite was observed at finer scales^20^. However, a deep understanding of the effect of habitat factors, especially interspecific interaction-linked habitat factors, on herbivore habitat selection or distribution, population density, physical characteristics and nutritional quality at regional and local scales are lacking in the literature.

Spatial distribution of wildlife depends on the spatial variation of resources, and distribution is rarely uniform^21^. Thus, on the local scale, wild animal resource selection might be varied due to spatial variation of resource distribution. However, although animal selection of resources varies with spatial scale^22^, important habitat factors that determine the fitness of a wild animal are expected to be selected at the highest or regional scale^23^. Finally, physical or nutritional pressure indicators may reflect the health status of a wildlife population^15^,^16^. In this study, we provided the following hypotheses: 1) ecological interaction chain responses of moose to sympatric interspecific interaction-linked habitat factors at the regional scale may exist; 2) moose may exhibit the general response (i.e., population density and environmental stress) to habitat factors and interactions with sympatric ungulates; and 3) then, moose habitat selection and environmental stress may be varied due to spatial heterogeneity driven by habitat factors or different interaction patterns with sympatric ungulates at the local scale.

## Results

Resource selection models suggested that moose had uniform preferences for some sympatric interspecific interaction-linked habitat factors at the regional scale (Extend Data Table 1). At the regional scale, the three habitat variables were entered into the moose resource selection model, which showed a similar preference for avoiding deep slopes (*p* < 0.05), maintaining a certain distance from mixed forest (*p* < 0.05), and the population density of moose (*p* < 0.05) were positive with resource selection (Extend Data Table 1). At the local scale, generalized linear models (GLM) for selection of food resources was positive (*p* = 0.07) in Shuanghe but it was negative in Nanwenghe (*p* < 0.05). For the interactions with roe deer occurrence probability, it was negative in Hanma (*p* < 0.05); however, those of both Nanwenghe (*p* < 0.05) and Zhanhe (*p* < 0.05) were crosscurrent with that of Hanma. For the distance to mixed forest, in both Hanma and Meitian, it was positive (*p* < 0.05) and negative in Zhanhe, (*p* < 0.05) (Extend Data Table 2). The number of model variables varied in six local sites (Extend Data Table 3), which showed that moose might have different requirements of food resource selection in the six local sites.

We used Five-fold Cross Validation for examining the model accuracy at local and regional scales, and we found the Area Under roc Curves (AUCs) of both local and regional models have predicable capacity and greater than 0.6 (Extend Data Table 4).

**Figure 1:**
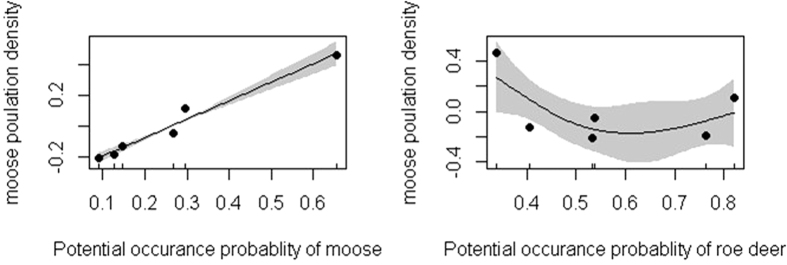
Relationships between potential occurrence probability of moose and moose population density (**a**); potential occurrence probability of roe deer and moose population density (**b**) at the regional scale. Maps were created using by R software (R Development Core Team, R i386 3.1.2; www.r-project.org).

**Figure 2:**
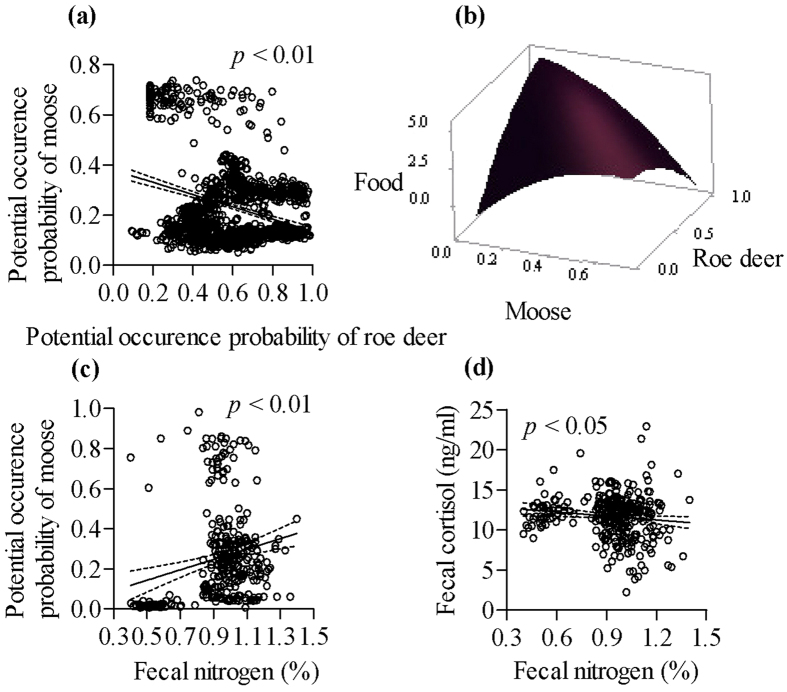
Relationships between potential occurrence probability of moose and roe deer (**a**), food availability with potential occurrence probability of moose and roe deer (**b**), moose fecal nitrogen with potential occurrence probability of moose (**c**), and moose fecal nitrogen with fecal cortisol (**d**) at the regional scale. Maps (Fig. 2b)were created using by Minitab (Minitab Statistical Software, Minitab 17.0; www.minitab.com); other maps were created using by Prism (GraphPad Prism, Prism 5.0; www.graphpad.com).

Our results showed that moose population density varied among local sites mainly because of different potential occurrence probability of moose at regional scale (*p* < 0.05) (Fig. 1a), which also showed that the habitat quality was important for improving moose population density. When examining the relationships between population density and potential occurrence probability of roe deer at regional scale (Fig. 1b), we found that the roe deer occurrence probability had the significant relationship with moose population density (*p* < 0.05) at coarse scale, it showed moose population density was influenced by roe deer. For interspecific interactions between moose and roe deer at finer scale, we found that the higher roe deer potential occurrence probability lead to lower moose potential occurrence probability (Fig. 2a). The interaction with moose and roe deer for food resources showed the different intensities of ecological separation among them, although both of them showed a preference for habitats with more abundant food (Fig. 2b), which suggested that moose and roe deer might avoid competition. Moose potential occurrence probability was positive with moose fecal nitrogen (Fig. 2c), which indicated that moose might select a habitat with higher diet quality. Finally, we found the moose fecal cortisol was negative with moose fecal nitrogen (Fig. 2d). Hence, the response of moose when there were interactions with roe deer was obvious at the regional scale.

**Figure 3:**
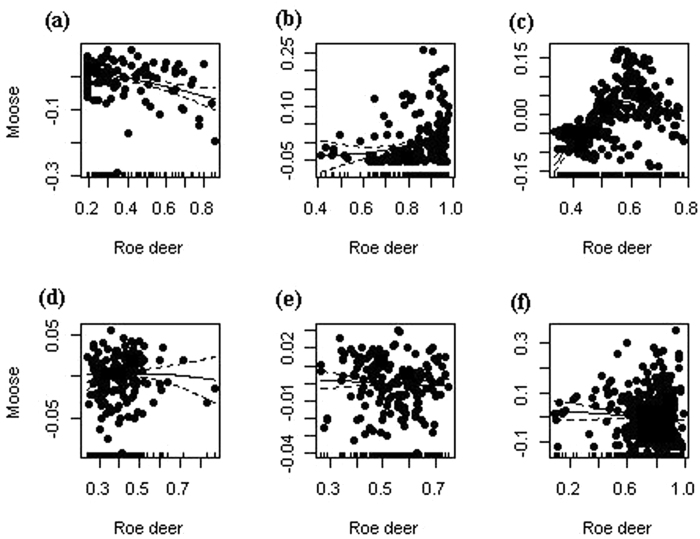
The nonlinear relationships between potential occurrence probability of moose and roe deer at local sites: Hanma (**a**), Shuanghe (**b**), Nanwenghe (**c**), Meitian (**d**), Mohe (**e**) and Zhanhe (**f**). Maps were created using by R software (R Development Core Team, R i386 3.1.2; www.r-project.org).

For analyzing the interaction patterns between moose and roe deer at the local scale, we found dissimilar relationships between moose and roe deer potential occurrence probability. They were the negative tendency in Hanma (Fig. 3a, *p* < 0.05), Mohe (Fig. 3e, *p* > 0.05) and Zhanhe (Fig. 3f, *p* > 0.05), which showed that moose and roe deer selected different habitats and existed in habitat segregation to a varying extent. The results indicated either that moose and roe deer had weak interactions or that there was a superiority of roe deer competition for moose at three local sites. However, there were adverse tendencies at Shuanghe (Fig. 3b, *p* < 0.05), which showed that moose and roe deer selected common habitat and that high habitat overlap existed, i.e., strong competition was found at this local site. Nanwenghe (Fig. 3c, *p* < 0.05) and Meitian (Fig. 3d, *p* > 0.05) were similar with normal distribution and hence, the two local sites showed complete competition between the moose and roe deer.

**Figure 4:**
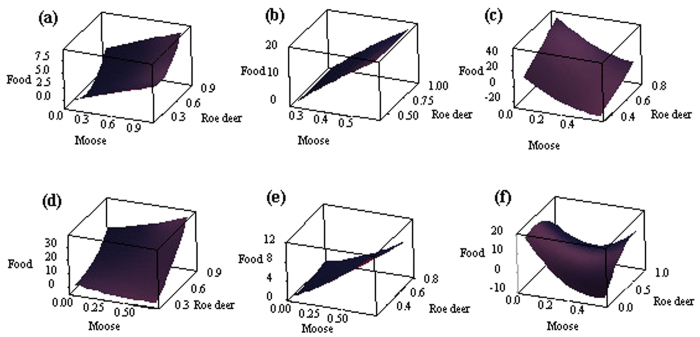
Relationships from the analysis of three-dimensional surface among food availability, potential occurrence probability of moose and roe deer at local sites: Hanma (**a**), Shuanghe (**b**), Nanwenghe (**c**), Meitian (**d**), Mohe (**e**) and Zhanhe (**f**). Maps were created using by Minitab (Minitab Statistical Software, Minitab 17.0; www.minitab.com).

Based on the three-dimensional surface analysis at the six local sites, we found that moose potential occurrence probability was positive with the food resource abundance and also that roe deer had the same tendencies as moose at Hanma (Fig. 4a); moose had positive tendency with food, and roe deer randomly selected food in Shuanghe (Fig. 4b) and Mohe (Fig. 4e). While roe deer potential occurrence probability was higher, moose preferred more abundant food resources in Mohe. Moose and roe deer had difference tendencies with food resources in Nanwenghe (Fig. 4c), Meitian (Fig. 4d) and Zhanhe (Fig. 4f).

**Figure 5:**
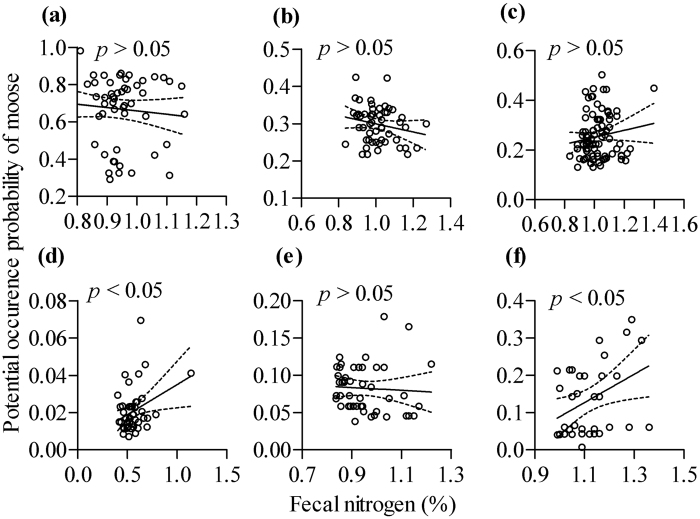
Relationships between potential occurrence probability of moose and moose fecal nitrogen at local sites: Hanma (**a**), Shuanghe (**b**), Nanwenghe (**c**), Meitian (**d**), Mohe (**e**) and Zhanhe (**f**). Maps were created using by Prism (GraphPad Prism, Prism 5.0; www.graphpad.com).

**Figure 6:**
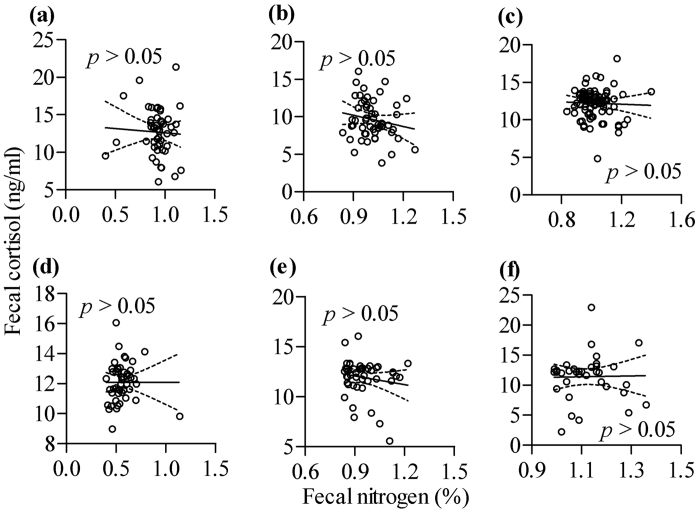
Relationships between moose fecal nitrogen and fecal cortisol at local sites: Hanma (**a**), Shuanghe (**b**), Nanwenghe (**c**), Meitian (**d**), Mohe (**e**) and Zhanhe (**f**). Maps were created using by Prism (GraphPad Prism, Prism 5.0; www.graphpad.com).

In addition, linear models for the relationships between moose potential occurrence probability and moose fecal nitrogen at each local site are shown in Fig. 5. The results suggested that at Nanwenghe (Fig. 5c, *p* > 0.05), Meitian (Fig. 5d, *p* < 0.05) and Zhanhe (Fig. 5f, *p* < 0.05), there was a positive tendency, but at Hanma (Fig. 5a, *p* > 0.05), Shuanghe (Fig. 5b, *p* > 0.05) and Mohe (Fig. 5e, *p* > 0.05), there was no significantly negative tendency. The differences in these local sites might be influenced by sympatric roe deer interaction patterns. We found dissimilar relationships between moose fecal nitrogen and fecal cortisol at each local site compared with those ecological relationships at regional scale (Fig. 6a–f, *p* > 0.05), which indicated that moose physiological response might be partly influenced by diet quality at the local scale.

## Discussion

**Figure 7:**
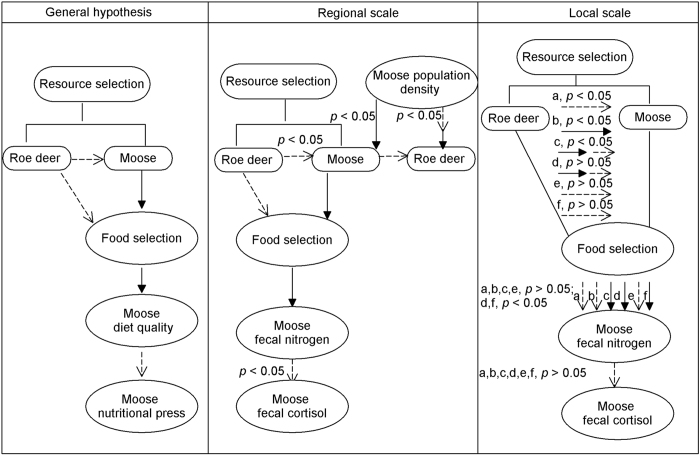
Response of moose population to habitat factors related to interaction with roe deer at regional and local scales. The moose and roe deer represent their potential occurrence probabilities, respectively. The solid line represents the positive effect, dotted line represents negative effect at the local scale: Hanma (**a**), Shuanghe (**b**), Nanwenghe (**c**), Meitian (**d**), Mohe (**e**) and Zhanhe (**f**). Maps were created using by Diagram Designer (Diagram Designer, Diagram Designer 1.28; logicnet.dk/DiagramDesigner/).

Based on the above results, we summarized a general ecological relationship chain of moose relating to interspecific interaction-linked habitat factors at the regional scale. Meanwhile, we also found some differences in responses of both moose habitat selection and environmental stress resulted from different interaction patterns with sympatric roe deer potential occurrence at the local scale. Different intensities of interspecific interaction might lead to different food resource availability, and then environmental stress. Furthermore, local heterogeneity in ecological-driven chains related to interspecific interaction was partly exhibited (Fig. 7).

### Effects of interspecific interaction

Interspecific interaction is considered one of the most important selective pressures affecting sympatric coexistence^24^. In the majority of moose habitats in China, large predators of herbivores have disappeared, and in this case in particular, resource competition between coexistence moose and roe deer was the most typical interspecific interaction^18^. A major goal in our study was to understand moose habitat selection and environmental stress as a response to interspecific interaction-linked habitat factors at both local and regional scales.

Interspecific interaction of sympatric species is thought to have an important effect on the abundance and distribution of each species^25^, which implies that competitive interactions may influence the occurrence probability of sympatric species^19^. Our results suggested that both moose potential occurrence probability and population density were influenced by sympatric roe deer potential occurrence probability. Coexistence of moose and roe deer is likely to influence moose resource selection patterns due to their similar foraging requirements^26^. Our results also showed that the relationships between moose and roe deer appear to be mainly regulated by variations in local food conditions. Hence, food resource selection of moose is likely to be determined by food nutrient quality in the absence of predators^12^. In this study, we found that moose trophic diet quality, estimated by moose fecal nitrogen, might be lower when moose potential occurrence probability declined, which implied that moose diet quality was influenced by interspecific interactions.

Population density is the fundamental driver of wildlife resource selection^7^ and interspecific interaction may also have an important effect in determining population density^27^. Our results showed that lower moose potential occurrence probability lead to lower moose population density, and the lower of roe deer occurrence probability resulted in the higher of moose population density. Fecal cortisol is thought to be the indicator of environmental stress of animal and several studies that have used this type of assessment to emphasize the need to carry out species-specific validations before using it to assess biological responses associated with stress^28^,^29^. Here, we also found that moose have a higher diet quality when there is evidence of higher moose potential occurrence probability, which might reduce moose stress at the regional scale. To some extent, our study indicated that moose food selection, diet quality, population density and environmental stress were a series of ecological relationships influenced by the interaction with sympatric roe deer distribution, which can form an ecological chain effect in the ecological system.

### Effects of scale and spatial heterogeneity

Ecological process, including resource selection, can occur at different spatial scales^22^. In this study, the moose resource selection model at the regional scale showed the same preference for avoiding abrupt slope, distance to mixed forest and higher population density. Local density of moose may also influence resource selection, and the distribution of individuals among mixed forest and slope^8^. Therefore, the regional model suggested that similar preferences for habitat variables but it also might cover the inter-population interaction and the role of food resource distribution. This result showed that moose distribution was partially affected by habitat characteristics at larger spatial scale^9^. Interspecific interaction between sympatric ungulates is stronger at finer scale than at coarse spatial scale^30^. At the six local sites, different interspecific interaction patterns between moose and roe deer appeared in the resource selection model. We also found differences at both regional and local scales of moose resource selections. The differences in both resource selection and responses of environmental stress were shown at different scales in this study (Fig. 7).

Spatial heterogeneity in relative availability of different habitat variables might result in the differences in resource selection among similar individuals^31^. A central feature of most species is adapting to local spatially heterogeneous environments^32^. This study indicated the presence of moose interacted with roe deer occurrence, the food selection of moose and distance to mixed forest held different preferences at the six local sites. A common trade-off faced by herbivores took place where the habitats provide the best foraging opportunities. For example, mixed forest can provide high ungulate food productivity and shelter^9^ and, to some extent, both moose and roe deer prefer selecting for this forest type. However, the distribution and abundance of main food resources often exhibit bio-geographical variation^33^. Habitat factors may influence the degree of diet overlap, which was directed by differences in interaction pressure^34^. Our regional results indicated that moose avoided competition with roe deer for food, but also the interactions showed different intensities of interspecific competitions at the six local sites (Fig. 3a–f) between moose and roe deer because of varying food resource availability (Fig. 4a–f). This reason may be that roe deer exhibited a greater capacity for coping with human disturbance and interspecific interactions^18^. Furthermore, our results also showed that the different intensities of interspecific interaction might result in different nutritional requirements and pressures. Consequently, spatial heterogeneity may be a basal driver in the ecological chain related to interspecific interactions.

Based on the above findings, future protection and management of moose populations and habitats should not only focus on the general ecological relationship dynamics of monitoring indicators of interspecific interaction-linked habitat factors and population environmental stress at the regional scale, but also those at the local scale. These findings might be useful for other sympatric mammal assembly conservation and management. In recent years, moose habitat was shrinking back towards the north and northwest of China, presumably due to global climate warming^35^,^36^,^37^,^38^. Managers should consider interspecific interaction-linked habitat factors and population environmental stress by regulating the density of roe deer populations and spatial distribution through long term and multiple spatial scales monitoring of the population, habitat quality and nutritional indicators.

## Methods

### Study area and data collection

Our study was conducted during the winters of 2011–2015. Moose mainly distribute in the Greater and Lesser Khingan mountain ranges of northeastern China. We selected the six local sites, i.e., Hanma, Shuanghe, Nanwenghe, Meitian, Mohe and Zhanhe, across the different geographical gradients within the current range of the moose (latitude 48°39′N–53°33′N, longitude 121°07′E–128°24′E) with intervals of more than 100 km among the six study sites (Extend Data Fig. 1).

We collected sympatric wild animal signs and habitat variables data by snow line transect survey in the field. The length of each line transect was more than 3 km. These lines were systematically distributed with intervals of 3 km covering each of the six local sites. We set up nine line transects within the Hanma (approximately covered 73.0 km^2^), 12 in Shuanghe (approximately covered 70.1 km^2^), 17 in Nanwenghe (approximately covered 198.6 km^2^), 15 in Meitian (approximately covered 92.7 km^2^), 23 in Mohe (approximately covered 212.8 km^2^), 20 in Zhanhe (approximately covered 197.9 km^2^). And we set up survey plots (10 m × 10 m) with intervals of 200 m measured by GPS handset along them. Furthermore, five small plots (2 m × 2 m) were laid out in each 100 m^2^ survey plot. One small plot was located in the center and the other four were at the corners of the 100 m^2^ plot, and we measured snow depth and the total number of moose staple (birch, *Betula spp*.; willow, *Salix spp*.; hazelnut, *Corylus spp*.; aspen, *Populus spp*.) and secondary (Mongolian oak, *Quercus spp*.; alder, *Alnus spp*.; lespedeza, *Lespedeza spp*.; rhododendron, *Rhododendron spp*.; pinus sylvestris, *Pinus sylvestris Linn*; spruce, *Picea asperata Mast*; tilia, *Tilia spp*.; larch, *Larix spp*.; elm, *Ulmus spp*.) food twigs in each small plot along the line transects.

We downloaded digital elevation model data and forest type data from the website (www.gcloud.com), anthropological data (village points and road polylines) and river polyline data from the forest type vector diagram of the six local sites. Furthermore, we extracted habitat covariates (Extend Data Table 5) based on the line transect survey using ArcGIS10.3.

We collected moose fecal samples as we walked along the line transects or following snow tracks at the six local sites. We collected a total of 320 fecal samples, including 49 in Hanma, 54 in Shuanghe, 87 in Nanwenghe, 48 in Meitian, 47 in Mohe and 36 in Zhanhe. After being dried and crushed, all of the fecal samples were analyzed for fecal nitrogen by Kjeldahl method^39^ with Kjeldahl^TM^ 8400 from FOSS. Fecal cortisol was determined using radioimmunoassay (RIA)^40^.

We used moose fecal samples for identifying individuals by non-invasive genetic capture and recapture methods (CMR)^41^, In the field sampling process, same individual fecal samples are often got in different places. According to the CMR statistical method, recording any one of them as the individual, other records can be regarded as the recapture of the individual^42^. There is a variety of software to calculate the population number of such samples, CAPWIRE^43^ is one of them. According to the probability of captured with the sampling objects the CAPWIRE software is divided into two models: the ECM (Even Capture Model) model and the TIRM (Two Innate Rates Model) model. In this paper, we use the CAPWIRE package in the R software to compute with two models, and use the bootstrap test of 10 000 to generate both models the 95% confidence interval (95% CI) for population number. And use the software’s Likelihood Ratio Test function to test the applicability of the sampled number distribution for each model. Based on the obtained *p* value, if a model test results *p* value significantly deviates from 0, then the sample number distribution follows the assumptions of the model. According to these, a suitable model is determined and the model is used to evaluate the result of population number. The estimated number of moose individuals was 55 (43–68) in Hanma, 28 (22–43) in Shuanghe, 47 (41–79) in Nanwenghe, 15 (14–18) in Meitian, 16 (12–19) in Mohe and 20 (17–24) in Zhanhe, then we assessed moose population density according to the size of survey area (Extend Data Table 6).

### Data analyses

We used a binomial distribution (wild animals signs based on the line transects, the pixel is 200 m, presence is 1; absence is 0) with a logit link function in generalized linear models (GLM or generalized linear mixed model (GLMM, random factor is the local site), which used all possible subsets of habitat covariates^44^. Interactions among sympatric ungulates are likely to influence moose resource selection patterns. Moose and roe deer have similar foraging requirements. Hence, we estimated the potential occurrence probability of other sympatric wild animals (Extend Data Table 5) by GLM with binomial distribution at the six local sites. We estimated the moose resource selection for habitat variables and potential occurrence probability of other sympatric mammal species through GLM and GLMM with binomial distribution at local and regional scales. When considering the resource selection of wild animals, both environmental and geographical variables should be treated equally^11^ and population density estimation was taken as the fundamental driver of wild animal resource selection^7^. We also considered moose population density as one variable of the six local sites in moose resource selection model (GLMM) at the regional scale.

Some habitat covariates were highly skewed, so we normalized them by standard transformations. We used a Pearson’s correlation matrix to identify problematic collinearities among habitat covariates (i.e., rs > 0.5)^45^. For correlated habitat covariates, we retained the covariates on behalf of a greater portion of the model deviance and ecological significance. These remainder covariates entered into the model. We used an information-theoretic approach to guide model development or selection^46^. We adjusted for small sample size and the minimum of the model, which used the difference of Akaike Information Criterion (AICc) to evaluate and choose the most parsimonious model at both local and regional scales, respectively. Finally, we found the most parsimonious model of moose considering habitat factors, as well as other sympatric species potential occurrence probability (Extend Data Table 3). We used k-fold cross validation to examine the most parsimonious model accuracy^47^ for local and regional scale models (Extend Data Table 4).

To reveal the response of moose to interspecific interaction-linked habitat factors at the regional scale, we linked moose population density to its and roe deer potential occurrence probability by Generalized Additive Model (GAM) at coarse scale; in addition, because a single density was applied across all samples in a given site, we used the mean of them in order to eliminate pseudoreplicated problems^48^. At the regional scale, we firstly linked roe deer and moose potential occurrence probability by linear models to find the interaction patterns between them; secondly, we used the three-dimensional surface analysis in order to reveal the moose and roe deer interactions related to food resources competition; thirdly, we linked the moose potential occurrence probability to its fecal nitrogen for revealing the relationship between habitat quality and diet quality; finally, we linked moose fecal nitrogen to fecal cortisol at finer scale. At the local scale, we linked roe deer to moose potential occurrence probability using GAM; for the difference of moose resource selection at six local sites, we revealed the two ungulate species food resource interaction patterns through three-dimensional surface analysis method among moose, roe deer potential occurrence probability and food availability; and linked moose potential occurrence probability to its fecal nitrogen, and linked its fecal nitrogen to its fecal cortisol by using linear models. The three-dimensional surface analysis was carried out using Minitab Statistical Software version 17 (Minitab, Inc. 2015. www.minitab.com). The model analysis of linear model (LM), GLM, GLMM and GAM were carried out using the mgcv and lme4 packages in R software (R Development Core Team, 2015. www.r-project.org).

All study was in accordance with the guidelines approved by The American Society of Mammalogists^49^. Our fieldwork and laboratory experiments were assessed and approved by Expert Committee of Feline Research Center of Chinese State Forestry Administration.

## Additional Information

**How to cite this article**: Bao, H. *et al*. Effects of interspecific interaction-linked habitat factors on moose resource selection and environmental stress. *Sci. Rep.* **7**, 41514; doi: 10.1038/srep41514 (2017).

**Publisher's note:** Springer Nature remains neutral with regard to jurisdictional claims in published maps and institutional affiliations.

## Supplementary Material

Supplementary Information
