# Supplementary material for: Effects of interspecific interaction-linked habitat factors on moose resource selection and environmental stress
Source: Sci Rep. 2017 Jan 27;7:41514. doi: 10.1038/srep41514 (PMC5269734; doi:10.1038/srep41514)

**Effects of** **interspecific interaction-linked habitat factors on moose resource selection and nutritional status**

Heng Bao^1^·John M. Fryxell^2^·Hui Liu^1^·Hongliang Dou^1^·Yingjie Ma^1^·Guangshun Jiang^1*^

^1^Feline Research Center of Chinese State Forestry Administration, College of Wildlife Resources, Northeast Forestry University, 26 Hexing Road, Harbin, Heilongjiang 150040, P.R. China. ^2^Department of Integrative Biology, University of Guelph, Guelph, Ontario, Canada, N1G 2W1.

**^*^** Corresponding author. E-mail: [jgshun@126.com](mailto:jgshun@126.com)

**Additional Supplementary Information:**

**Extend Data Table 1**｜Parameters of the most parsimonious model of generalized liner mixed model (GLMM) at regional scale, the sample size is 200 m. Different sites were as random effect variables in this model.

| Var. | Coeff. | SE | Sig. | 95% CI | |
| --- | --- | --- | --- | --- | --- |
| (Intercept) | -2.39 | 0.13 | **0.00** | -2.65 | -2.15 |
| Slope | -0.16 | 0.08 | **0.04** | -0.33 | -0.01 |
| Distance to mixed | 0.40 | 0.07 | **0.00** | 0.26 | 0.54 |
| Density of moose | 6.36 | 0.51 | **0.00** | 5.37 | 7.38 |

**Extend Data Table 2**｜Parameters of the most parsimonious models of general liner model (GLM) in 6 local sites, the sample size is 200 m .

| Sites | Var. | Coeff. | SE | Sig. | 95% CI | |
| --- | --- | --- | --- | --- | --- | --- |
| Hanma | Intercept | 2.15 | 0.48 | **0.00** | 1.26 | 3.14 |
|  | Roe deer | -4.01 | 1.18 | **0.00** | -6.43 | -1.79 |
|  | Distance to mixed | 0.96 | 0.24 | **0.00** | 0.51 | 1.45 |
|  | Distance to river | -0.40 | 0.21 | 0.06 | -0.82 | 0.02 |
| Shuanghe | Intercept | -0.78 | 0.17 | **0.00** | -1.13 | -0.46 |
|  | Food | 0.29 | 0.16 | 0.07 | -0.03 | 0.61 |
| Nanwenghe | Intercept | -3.47 | 0.69 | **0.00** | -4.87 | -2.15 |
|  | Roe deer | 4.37 | 1.21 | **0.00** | 2.03 | 6.93 |
|  | Food | -0.37 | 0.16 | **0.02** | -0.72 | -0.08 |
| Meitian | Intercept | -2.59 | 0.35 | **0.00** | -3.37 | -1.97 |
|  | Snow | 0.79 | 0.35 | **0.02** | 0.14 | 1.51 |
|  | Distance to mixed | 0.86 | 0.24 | **0.00** | 0.40 | 1.36 |
|  | Distance to swamp | -0.58 | 0.30 | **0.05** | -1.25 | -0.04 |
| Mohe | Intercept | -2.61 | 0.33 | **0.00** | -3.34 | -2.02 |
|  | Aspect | 0.60 | 0.29 | **0.04** | 0.05 | 1.21 |
|  | Food | 0.46 | 0.22 | **0.04** | 0.00 | 0.90 |
|  | Slope | -0.52 | 0.34 | 0.13 | -1.26 | 0.08 |
| Zhanhe | Intercept | -4.34 | 1.19 | **0.00** | -6.75 | -2.05 |
|  | Distance to mixed | -0.78 | 0.20 | **0.00** | -1.18 | -0.41 |
|  | Distance to needle | -0.71 | 0.29 | **0.02** | -1.32 | -0.16 |
|  | Elevation | -0.66 | 0.27 | **0.01** | -1.19 | -0.14 |
|  | Food | -0.45 | 0.34 | 0.19 | -1.23 | 0.04 |
|  | Roe deer | 2.97 | 1.50 | **0.05** | 0.07 | 5.99 |

**Extend Data Table 3**｜Number of model parameters (K), Akaike’s Information Criterion (AIC) scores of the most supported GLM (family = binomial) by stepwise regression for moose resource selection of regional sites, and GLMM (family is binomial, random effect is group).

| Site | Logistic model | K | AIC |
| --- | --- | --- | --- |
| Hanma | Roe deer + Distance to mixed + Distance to river | 3 | 148.7 |
| Shuanghe | Food | 1 | 210.24 |
| Nanwenghe | Roe deer + Food | 2 | 350.98 |
| Meitian | Snow depth + Distance to mixed + Distance to swamp | 3 | 96.38 |
| Mohe | Aspect + Food + Slope | 3 | 99.38 |
| Zhanhe | Roe deer + Distance to mixed + Food + Distance to needle + Elevation | 5 | 285.13 |
| All sites | Slope + Distance to mixed + Density of moose | 3 | 1256.9 |

**Extend Data Table 4**｜Five-fold cross validations for GLM and GLMM, at regional and local scales.

| Site | AUC | Average of AUC | CV AUC |
| --- | --- | --- | --- |
| Hanma | 0.77 | 0.75 | 0.01 |
| Shuanghe | 0.61 | 0.60 | 0.04 |
| Nanwenghe | 0.67 | 0.67 | 0.03 |
| Meitian | 0.80 | 0.80 | 0.02 |
| Mohe | 0.72 | 0.72 | 0.03 |
| Zhanhe | 0.73 | 0.73 | 0.04 |
| All sites | 0.72 | 0.72 | 0.01 |

**Extend Data Table 5**｜Habitat variables tested for moose resource selection in 6 local sites.

| Habitat factor | Description of habitat factor | Data type | Unit |
| --- | --- | --- | --- |
| Snow depth | Measured five times in survey plots (10m×10m) with intervals of 200m  (measured by GPS handset) on line transect | Continuous | cm |
| Aspect | Aspect grid with 30m resolution | Continuous | degree |
| Elevation | Elevation grid with 30m resolution | Continuous | m |
| Slope | Slope grid with 30m resolution | Continuous | degree |
| Distance to needle | Distance from the central point of each 200m pixel to the needle evergreen forest | Continuous | m |
| Distance to broadleaf | Distance from the central point of each 200m pixel to the broad-leaved deciduous forest | Continuous | m |
| Distance to shrub | Distance from the central point of each 200m pixel to the shrub | Continuous | m |
| Distance to swamp | Distance from the central point of each 200m pixel to the swamp | Continuous | m |
| Distance to mixed | Distance from the central point of each 200m pixel to the mixed broad-leaved and needle-leaved forest | Continuous | m |
| Distance to river | Distance from the central point of each 200m pixel to the river | Continuous | m |
| Distance to road | Distance from the central point of each 200m pixel to the road | Continuous | m |
| Distance to village | Distance from the central point of each 200m pixel to the village in the 5km buffer of study region | Continuous | m |
| Staple food | Counted the number of moose staple food twigs in survey plots (10m×10m) with intervals of 200m (measured by GPS handset) on line transect | Continuous | N/m^2^ |
| Secondary food | Counted the number of moose secondary food twigs in survey plots (10m×10m) with intervals of 200m (measured by GPS handset) on line transect | Continuous | N/m^2^ |
| Food | The total number of food twigs in survey plots (10m×10m) with intervals of 200m (measured by GPS handset) on line transect | Continuous | N/m^2^ |
| Roe deer | Potential occur probability of roe deer from GLM model | Continuous | ratio |
| Wild boar | Potential occur probability of wild boar from GLM model | Continuous | ratio |
| Hare | Potential occur probability of hare from GLM model | Continuous | ratio |
| Lynx | Potential occur probability of lynx from GLM model | Continuous | ratio |
| Sable | Potential occur probability of sable from GLM model | Continuous | ratio |
| Red deer | Potential occur probability of red deer from GLM model | Continuous | ratio |
| Density of moose | Population density of moose by CMR | Continuous | N/Km^2^ |

**Extend Data Table 6**｜Moose population density identified by non-invasive genetic capture and recapture methods (CMR).

| Site | Area (km^2^) | Moose individuals | Population density (individuals/km^2^) | 95% CI | |
| --- | --- | --- | --- | --- | --- |
|  |  |  |  | Lower | Upper |
| Hanma | 73.01 | 55 | 0.75 | 0.59 | 0.93 |
| Shuanghe | 70.11 | 28 | 0.40 | 0.31 | 0.61 |
| Nanwenghe | 198.62 | 47 | 0.24 | 0.21 | 0.40 |
| Meitian | 92.71 | 15 | 0.16 | 0.15 | 0.19 |
| Mohe | 212.79 | 16 | 0.08 | 0.06 | 0.09 |
| Zhanhe | 197.94 | 20 | 0.10 | 0.09 | 0.12 |

**Extend Data Figure 1**｜Six study local sites and moose distribution area in northeast China. Maps were created using ArcGIS software by Esri (Environmental Systems Resource Institute, ArcGIS 10.2; www.esri.com), the layers included the Chinese map (1:250000 fundamental geographic data, download in www.ngcc.cn), elevation (the grid size is 90 m, download in srtm.csi.cgiar.org) and moose distribution area ^35^ in the map.


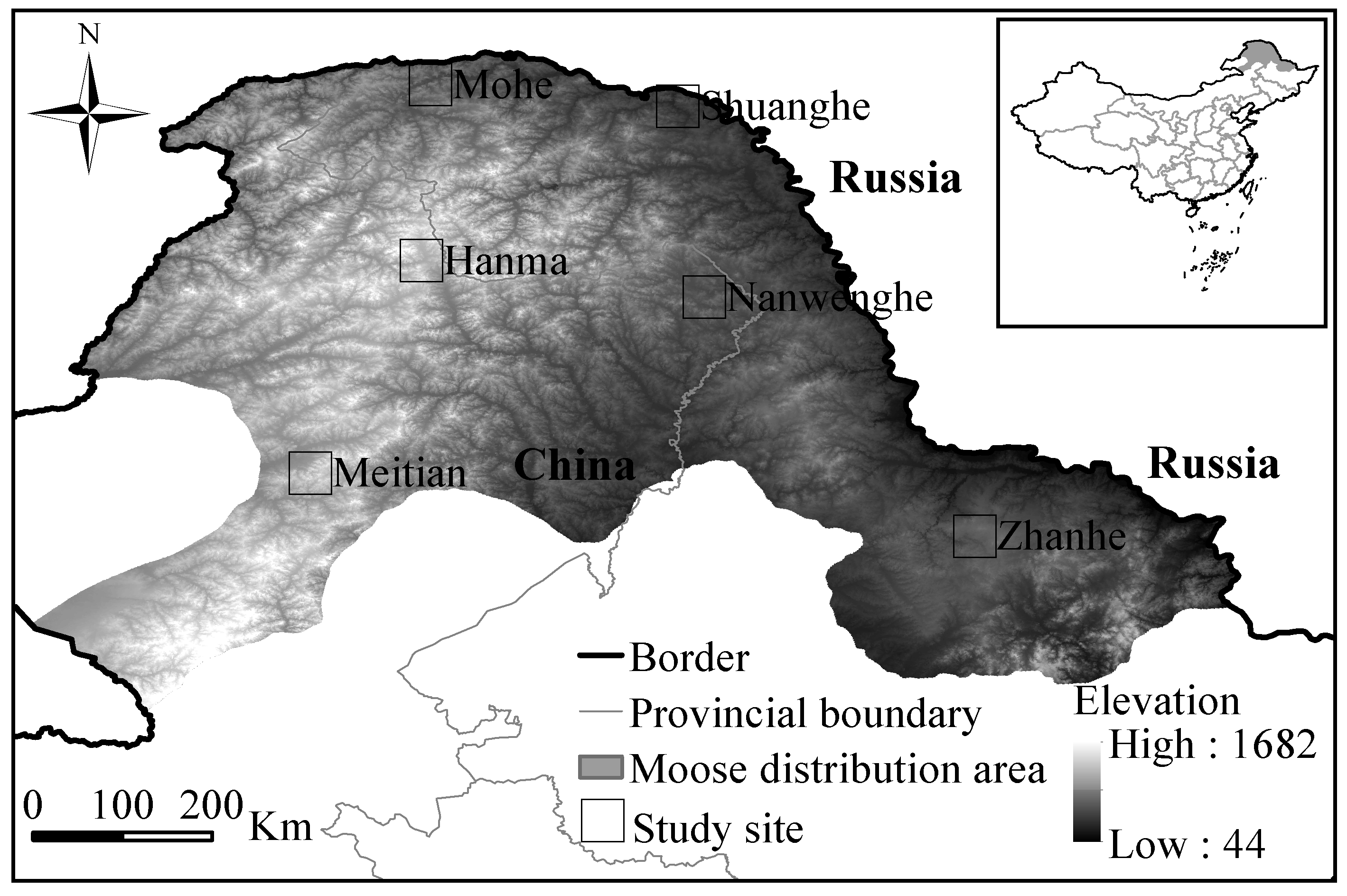

Supplement: Supplementary Information [file srep41514-s1.doc]
